# Coralline algae (Rhodophyta) in a changing world: integrating ecological, physiological, and geochemical responses to global change

**DOI:** 10.1111/jpy.12262

**Published:** 2015-01-23

**Authors:** Sophie J. McCoy, Nicholas A. Kamenos

**Affiliations:** ^1^ Department of Ecology and EvolutionThe University of Chicago1101 E. 57th StreetChicagoIllinois60637USA; ^2^ School of Geographical and Earth SciencesUniversity of GlasgowUniversity AvenueGlasgowG12 8QQUK; ^3^ Present address: Plymouth Marine LaboratoryProspect Place, The HoePlymouthPL1 3DHUK

**Keywords:** calcification, climate change, coralline algae, crustose coralline algae, ecology, ecosystem services, ocean acidification, paleoclimate, paleoclimate proxies, photosynthesis, physiology

## Abstract

Coralline algae are globally distributed benthic primary producers that secrete calcium carbonate skeletons. In the context of ocean acidification, they have received much recent attention due to the potential vulnerability of their high‐Mg calcite skeletons and their many important ecological roles. Herein, we summarize what is known about coralline algal ecology and physiology, providing context to understand their responses to global climate change. We review the impacts of these changes, including ocean acidification, rising temperatures, and pollution, on coralline algal growth and calcification. We also assess the ongoing use of coralline algae as marine climate proxies via calibration of skeletal morphology and geochemistry to environmental conditions. Finally, we indicate critical gaps in our understanding of coralline algal calcification and physiology and highlight key areas for future research. These include analytical areas that recently have become more accessible, such as resolving phylogenetic relationships at all taxonomic ranks, elucidating the genes regulating algal photosynthesis and calcification, and calibrating skeletal geochemical metrics, as well as research directions that are broadly applicable to global change ecology, such as the importance of community‐scale and long‐term experiments in stress response.

**Abbreviations**
CaCO_3_: calcium carbonate
CCA: crustose coralline algae
CO_2_: carbon dioxide
CO_3_^2−^: carbonate
DIC: dissolved inorganic carbon
HCO_3_^−^: bicarbonate
OA: ocean acidification
PAR: photosynthetically active radiation
SST: sea surface temperature

Coralline algae (Corallinales and Sporolithales, Corallinophycidae, Rhodophyta) are receiving renewed attention across the ecological and geological sciences as important organisms in the context of global environmental change, especially ocean acidification (OA). In addition to their important functional roles in ecological systems across latitudes and habitat types (e.g., reef frameworks, Adey 1998, Chisholm 2000, carbonate (CO_3_^2−^) production, Bosence 1980, foundational species, Steneck and Dethier 1994, larval settlement, Daume et al. 1999, fish nurseries, Kamenos et al. 2004a), coralline algae are increasingly used as paleoecological proxies (e.g., Cabioch et al. 1999, Braga and Aguirre 2001, Perry 2001, Aguirre et al. 2007) and accurate paleoenvironmental recorders (e.g., Halfar et al. 2000, Kamenos 2010, Williams et al. 2011), thus providing a valuable mechanism for contextualizing recent oceanic changes.

Coralline diversification reveals the ability of this group to colonize a wide range of light, temperature, and energy conditions and to remain chief components of benthic marine communities through considerable fluctuations in temperature and light over geologic time (Aguirre et al. 2000). Much is known about coralline algal ecology and physiology, despite the great variety in ecological forms and cryptic diversity emerging from molecular studies. Here, we point the reader to previous reviews of the basic ecology and physiology of coralline algae (Table 1) and focus on new insights into the potential responses of coralline algae to environmental change at different scales, including responses of physiology, skeletal mineralogy, ecology, and ecosystem services.

**Table 1:** Summary of previous reviews on the subject of coralline algae published in the last 40 years

Discipline									Growth form		Latitude		Timescale		Reference
Life history	Physiology	Ecology	Biogeography	Carbonate production	Calcification	Phylogenetics	Taxonomy	Sclerochronology	Geniculate	Nongeniculate	Tropical	Temperate	Modern	Paleo	Citation
×		×	×	×			×			×	×	×	×		Littler 1972
							×							×	Adey and Macintyre 1973
	×	×				×	×		×	×	×	×	×	×	Johansen 1981
		×								×	×	×	×	×	Steneck 1983
		×	×							×	×	×	×	×	Bosence 1983
				×	×						×		×		Littler and Littler 1984
		×	×							×	×	×	×	×	Steneck 1985
		×	×							×	×	×	×	×	Steneck 1986
×	×	×	×	×	×		×		×	×	×	×	×		Woelkerling 1988
							×							×	Aguirre et al. 2000
										×	×	×	×		Foster 2001
	×									×		×	×		Wilson et al. 2004
		×		×	×					×	×	×	×		Nelson 2009
×	×	×		×	×			×		×		×	×	×	Adey et al. 2013
		×		×				×		×	×	×	×	×	Foster et al. 2013

## Coralline Algal Ecology

### Nongeniculate (crustose and rhodolith forms)

**Figure 1:**
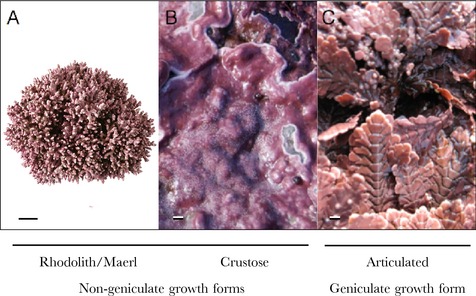
Examples of (A) rhodo‐lith (maerl), (B) crustose, and (C) geniculate growth forms of red coralline algae. Scale bars are 10 cm, 1 cm, and 5 mm, respectively. *Source*: (A) Photo by N.A. Kamenos, (B and C) photos by S.J. McCoy.

Nongeniculate coralline algae, or coralline algae lacking noncalcified articulations (genicula) between calcified segments (Fig. 1, A and B), are some of the most abundant organisms throughout the hard‐bottom marine photic zone (Adey and Macintyre 1973, Steneck 1986). This group includes crustose and rhodolith (or maerl) morphologies (Foster 2001). Nomenclature of free‐living forms is often inconsistent in the literature, which describes “coatings,” “gravels,” “rhodolites,” and most commonly “maerl” and “rhodoliths” (Steneck 1986). This terminology can be confusing, given that several species of nongeniculate coralline algae have been observed within an individual rhodolith or coated pebble (Basso 1998, Yabur‐Pacheco and Riosmena‐Rodríguez 2006). We thus refer to all forms not attached to hard‐bottom substratum or other macroalgae (including coralline algae) as rhodoliths, following the nomenclature of Foster (2001). Correspondingly, we define the term crustose coralline to refer to all forms that grow roughly radially on hard substrates and exhibit determinate thickness <1 cm. Many nongeniculate species are thought to exist in both rhodolith and crustose forms. However, we will occasionally separate our discussion of these two morphological groups due to some important differences in ecology and ecosystem services.

Nongeniculate coralline algae can be found on any hard substrate where light penetrates (Bosence 1983). They thrive in areas of moderate disturbance and often dominate in areas of high stress and disturbance potential where many other macrophytes are absent (Steneck 1986, Dethier 1994). This includes areas of high herbivory, wave action, sand scour, and low productivity potential such as the low photic zone, shaded understories of large macrophyte beds, and the intertidal zone (Kendrick 1991, Dethier 1994, Steneck and Dethier 1994, Dethier and Steneck 2001). Crustose forms often cover a high proportion of primary space despite a relatively flat morphology that makes them easy to overgrow (Dethier and Steneck 2001). Such areas are referred to as crustose coralline carpets (Paine 1984).

Rhodoliths are a morphologically diverse group of nongeniculate coralline algae, shaped like spheres, branching twigs, or fans and ranging from roughly 1–100 cm in size (Foster et al. 2013). Rhodolith beds tend to form on fairly level bottoms that have sufficient, but often low light, and occur in areas with moderate water motion and high bioturbation to prevent the burial of rhodoliths in sediment (Steller and Foster 1995, Connell 2003, Wilson et al. 2004, Harrington et al. 2005). Unlike crustose coralline carpets, rhodolith beds form in the absence of intense water movement, which could scatter or bury slow‐growing rhodoliths (Nelson 2009, Foster et al. 2013). Rhodolith beds can range several square kilometers in tropical and temperate settings (Foster 2001, Nelson 2009, Amado‐Filho et al. 2012, Foster et al. 2013), and therefore play a significant role in calcium carbonate (CaCO_3_) production on continental shelves (Amado‐Filho et al. 2012).

### Geniculate (articulated forms)

Geniculate or articulated coralline algae consist of an algal frond growing from a basal crust. The morphology of basal crusts varies among species and individuals, and can be either extensive or appear hidden beneath the frond. Geniculate corallines are named for the noncalcified joints (genicula) that occur between the larger calcified segments (intergenicula) in an upright frond, allowing it to flex with water movement (Fig. 1C). Geniculate corallines, like nongeniculate corallines and other noncalcified macroalgae, exhibit strong patterns of zonation throughout intertidal and subtidal zones depending upon their light, desiccation, and grazing tolerances (Padilla 1984, Martone 2010, Guenther and Martone 2014).

### Coralline algae illustrate ecological models of persistence

Primary substrate in the photic zone is highly contested, and thus coralline algae compete with each other as well as with fleshy and filamentous macroalgae and microalgae. Fast‐growing (up to 20 mm · year^−1^) nongeniculate and geniculate corallines are typically early colonizers and become replaced by slow‐growing, thicker, or branched crusts (Padilla 1981, Steneck 1986, Matsuda 1989) or noncalcified algal turfs (Kendrick 1991). Competitive interactions and susceptibility to herbivory among coralline algae have been well documented for many common species of both nongeniculates and geniculates, particularly in the Northeast Pacific (Paine 1980, 1984, Steneck 1986, Steneck et al. 1991, Dethier 1994, Steneck and Dethier 1994, Dethier and Steneck 2001). While the dominance structure is generally hierarchical and dictated by thallus thickness, edge morphology, and growth rate, reversals in the competitive hierarchy are common and typically mediated by herbivores (Paine 1984, Steneck et al. 1991). A particular species' competitive ability thus depends on its growth strategy and its resistance to grazing.

Nongeniculate corallines have both competitive and positive (facilitative) relationships with macroalgae. For example, many temperate nongeniculate corallines inhabiting the intertidal or shallow subtidal depend on shading by the macro‐algal canopy, while they also compete for light and holdfast space with large macrophytes (Paine 1980, 1984, Irving et al. 2004, 2005). Filamentous macroalgae may grow epiphytically on the crust surface (Figueiredo et al. 1996). Nongeniculate corallines have two primary mechanisms for the removal of epiphytic organisms from their surface: epithallial sloughing to shed surface cells and depending on herbivores to graze epiphytes off the thallus surface. During epithallial sloughing, an individual typically loses the uppermost layer of cells from its epithallus (Johnson and Mann 1986, Pueschel and Keats 1997, Figueiredo et al. 2000), though some species are “deep‐layer” sloughers, shedding below the layer of actively growing (meristematic) cells (Keats et al. 1993). An alternative hypothesis for the ecological function of epithallial sloughing is that constant sloughing leads to a thin thallus, which is correlated with faster growth and stronger attachment (Keats et al. 1994).

### Trophic interactions

Coralline algae can generally have both positive and negative interactions with grazers. Nongeniculate corallines benefit from low levels of herbivory (Steneck 1983, 1986), and grazer presence may even stimulate local productivity of coralline crusts (Wai and Williams 2005). In addition to sea urchins (Echinoidea), two molluscan groups are able to graze coralline algae. Limpets (Patellacea) and chitons (Polyplacophora) have several convergent adaptations for grazing hard substrates: strong buccal muscles, unique dentition, and a heavy silicate and iron mineral coating on their teeth (Steneck 1983). In tropical areas, common grazers also include fish. In return, coralline algae are particularly well adapted to withstand grazing with calcified thalli and conceptacles (Steneck 1985).

Crustose coralline communities exhibit what is called consumer‐mediated coexistence. The presence of grazers can overturn competition hierarchies by favoring grazer‐resistant species over fast growers. Herbivore‐mediated reversals slow the competitive exclusion of one species by another and are therefore important to the long‐term persistence of nongeniculate coralline species diversity (Paine 1984, Steneck et al. 1991, Dethier and Steneck 2001). This has been best documented experimentally in temperate intertidal and shallow subtidal systems, but is likely an important process globally given the high grazing rates documented in warmer‐water systems (Hay 1997). Heavy grazing can also induce morphological change in coralline algae (Maneveldt and Keats 2008), affecting algal competitive interactions, many of which are based on morphological traits such as thallus thickness and lateral growth rates (Paine 1984, Dethier and Steneck 2001, Maneveldt and Keats 2008). The basal crusts of geniculate corallines may compete for space with crustose species, but tend to be poor competitors due to their thinness (Paine 1984) and likely persist due to high colonization rates (Padilla 1981). Successional patterns in coralline algae have been well summarized by Steneck (1986) as a slow replacement of thinner, unbranched morphologies to be replaced by thicker and/or branched species.

### Secondary metabolites

Marine algae produce an array of secondary compounds (also referred to as secondary metabolites). A variety of physiological and ecological functions exist even for the same compound (reviewed in Hay 1997, 2009), primarily competitive interactions (Rasher and Hay 2010, Andras et al. 2012) and grazer deterrence (Norris and Fenical 1982, Faulkner 1984, Rasher and Hay 2014). These compounds differ in mechanisms of grazer deterrence and toxicity, but are generally thought to reduce palatability, digestibility, or nutrition of algal tissue, or to be toxic through effects on the nervous system or cardiac functions (Van Alstyne 1988). Anti‐grazer compounds often act as anti‐fouling agents and potentially reduce microbial pathogens (Schmitt et al. 1995). Coralline algae use a variety of chemical compounds to deter epiphytes, typically fatty acids that act as algal spore lytic agents (Figueiredo et al. 1997, 2000, Kim et al. 2004, Luyen et al. 2009). The degree of allelopathy depends not only on the coralline algae but also on the identity of its epiphyte (Bôas and Figueiredo 2004).

A large proportion of research in this area has focused on the role of dimethylsulphoniopropionate (DMSP) in coralline algae. DMSP is a secondary metabolite common in many marine algae that has been identified as a cryoprotectant (Karsten et al. 1996), an antioxidant (Sunda et al. 2002), and a possible grazer defense compound (Van Alstyne and Houser 2003, Lyons et al. 2010). DMSP has been detected at high concentrations in temperate rhodolith beds comprised of primarily *Lithothamnion glaciale* and *Phymatolithon calcareum*, both in algal tissue and in the water column (Kamenos et al. 2008a), likely functioning to combat oxidative stress (Rix et al. 2012). No change in DMSP concentrations has been documented in response to stable OA scenarios in *L. glaciale*, however, DMSP concentrations increase in response to sudden pH change, leading to epithallial damage (Burdett et al. 2012a). On a coral reef flat, the lightly calcified *Amphiroa* sp. increases DMSP concentrations to maintain metabolic function during periods of low CO_3_^2−^ saturation state (Burdett et al. 2013). Overall, DMSP production in coralline algae is slow process occurring at timescales of hours to days probably reflecting the energetic cost of its production (Rix et al. 2012, Burdett et al. 2013).

### Interactions in a changing ocean

As the competitors of coralline algae (other coralline and fleshy algal species) and herbivores (primarily calcified echinoderms and mollusks in temperate areas) may have differential responses to OA, it is imperative that the responses of communities be assessed to disentangle direct from indirect effects of acidification. Ecologically important parameters such as growth rate and thallus thickness are directly related to CaCO_3_ content and calcification rates. It is, therefore, not surprising that OA has been found to affect ecological interactions through effects on growth (Gao et al. 1993, Martin and Gattuso 2009, Ries et al. 2009, Ragazzola et al. 2012, 2013, Cornwall et al. 2013a, Egilsdottir et al. 2013, Kamenos et al. 2013, Noisette et al. 2013a,b, Kato et al. 2014) and both large‐ and fine‐scale morphology (Ragazzola et al. 2012, 2013, McCoy 2013, Kato et al. 2014, McCoy and Ragazzola 2014) as CaCO_3_ production becomes more costly. Examples of this include altered competitive interactions among coralline algae (McCoy and Pfister 2014), between coralline algae and noncalcified algae (Jokiel et al. 2008, Kuffner et al. 2008, Porzio et al. 2011, Kroeker et al. 2013), and between coralline algae and grazers (McCoy and Pfister 2014).

A high‐CO_2_ environment will especially affect ecological dynamics between coralline and noncalcified algae as the energetic cost of calcification increases. High acidity favors recruitment of fleshy algae over coralline algae (Kuffner et al. 2008, Kroeker et al. 2013), which will lead to an escalation of competition between coralline and nonepiphytic fleshy algae. Noncalcified or fleshy algae can benefit from elevated HCO_3_^−^ availability for faster photosynthetic growth without any associated negative responses of calcified tissue to lower CaCO_3_ saturation states that will simultaneously affect coralline algae (Jokiel et al. 2008, Kuffner et al. 2008, Porzio et al. 2011, Hofmann et al. 2012, Kroeker et al. 2013). This mechanism will also affect interactions between coralline and epiphytic fleshy algae. The primary mechanisms of epiphyte control involve sloughing or grazing, both of which cause loss of calcified growth, and are likely to become energetically costlier as acidification continues.

## Ecosystem Services

### Tropical systems

Despite their global distribution and importance, coralline algae are perhaps most commonly recognized for their ecological services in tropical settings. Coralline algae provide calcified cement between coral heads, and can be primary reef builders (Setchell 1926, Bak 1976, Adey 1978) that provide settlement substrate for other organisms (Gherardi and Bosence 1999) and physical frameworks (Nelson 2009). In addition, coralline algae in rhodolith beds can play a physical, stabilizing role that permits coral settlement and establishment of coral reefs over geologic timescales (Tierney and Johnson 2012).

Tropical nongeniculate coralline algae promote local biodiversity. As early colonizers, nongeniculate coralline algae may either inhibit or enhance recruitment of other individuals to the community. Coralline algae are typically thought of as enhancing recruitment or triggering larval metamorphosis of other species by providing chemical cues (Morse et al. 1979, 1988, Morse and Morse 1984, Johnson et al. 1991, Johnson and Sutton 1994, Figueiredo et al. 1997, O'Leary et al. 2012) or by providing a suitable attachment substrate or sufficient structural heterogeneity. Species‐specific colonization or induction cues may also, or instead, be associated with bacteria growing on the coralline algal surface, which are shed with algal cells during sloughing (Johnson et al. 1991, Johnson and Sutton 1994, Huggett et al. 2006).

These mechanisms are crucial to the diversity of tropical and temperate invertebrate communities and may be subject to change as the ocean environment changes. For example, under elevated seawater temperatures only 2°C–4°C above mean maximum seawater temperatures, the nongeniculate coralline *Neogoniolithon fosliei* experienced a large shift in the structure of its surface microbial community as well as its ability to induce coral larval metamorphosis at the elevated temperature (Webster et al. 2011). In another example, settlement of the coral *Acropora millepora* revealed potential changes in coral recruitment in response to OA; coral larvae increasingly avoid one of their preferred CCA substrates with rising *p*CO_2_ (Doropoulos et al. 2012).

Grazing pressure plays a role in coralline algal ecosystem services, as well. The facilitative relationship between coralline algal cover and settlement of reef invertebrates is susceptible to fishing‐induced trophic cascades, in which fishing increases urchin populations, which reduces cover of nongeniculate coralline algae and can thus be linked to reduced coral recruitment (O'Leary and McClanahan 2010, O'Leary et al. 2012). The reduction in coralline algal cover is exacerbated by reduced grazing by herbivorous fish in overfished regions, which favors the growth of fleshy algae over coralline algae (Belliveau and Paul 2002).

A major concern as OA intensifies has been on the impact of these reef ecosystem services as calcification becomes more difficult in coralline algae themselves. Tropical rhodolith beds are major players in the global carbon cycle through the production of CaCO_3_ sediment. In shallow reefs, for example, some species produce up to 9.1 g CaCO_3_ · m^−2^ · d^−1^ (Chisholm 2000), and 0.9–5 g organic carbon (net) planar · m^−2^ · d^−1^ (Chisholm 2003). The most expansive rhodolith bed sits off the coast of eastern Brazil on the Abrolhos Shelf, and extends 20,900 km^2^. In this bed, mean CaCO_3_ production is 1.07 kg · m^−2^ · year^−1^, totaling 0.025 Gt · year^−1^ (Amado‐Filho et al. 2012).

Over geologic timescales, the saturation states of calcite and aragonite have affected sediment production in tropical regions (Ries 2006a, 2009). Experimentally, lower calcification and primary production rates and reductions in tissue mass of *Halimeda*,* Penicillus*, and *Udotea* were observed under lower saturation states, suggesting that calcification may in fact promote photosynthesis through release of CO_2_ or H^+^ ions (Ries 2009). Reduced rates of primary production may have been aggravated by reduced coralline algal tissue mass or height of algal reefs (Ries 2009).

Coralline algae typically accrete high‐Mg calcite, or dolomite, skeletons. The presence of dolomite (Mg_0.5_Ca_0.5_CO_3_) in the nongeniculate corallines *Porolithon onkodes* and *Porolithon pachydermum* decreased the dissolution rate of coralline thalli by 6–10 times (Nash et al. 2012). Indeed, as the proportion of dolomite increases with acidity as other CO_3_^2−^ minerals dissolve out, this mechanism may indicate an optimistic future for the continued role of coralline algae as reef stabilizers (Nash et al. 2012). It is important to note, however, that calcification studies on the temperate intertidal geniculate *Corallina elongata* (Egilsdottir et al. 2013) and subtidal nongeniculate *L. glaciale* (Kamenos et al. 2013) have found that individuals raised under higher *p*CO_2_ incorporate a lower proportion of Mg^2+^/Ca^2+^ during calcification. This finding does not necessarily contradict the former, as dissolution over the longer term may ultimately favor the preservation of dolomite.

### Temperate, Subarctic, and Arctic systems

**Figure 2:**
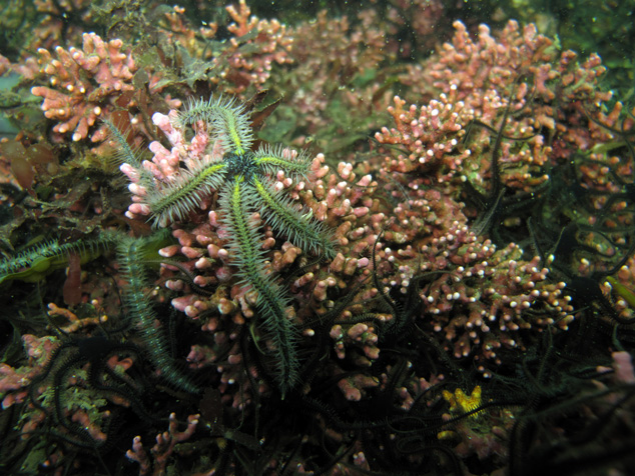
Temperate, subtidal *Lithothamnion glaciale* rhodolith bed off west coast of Scotland. Photo by N. Kamenos.

Rhodolith beds provide important hard substrate for colonization of other marine algae and invertebrates (Fig. 2; Kamenos et al. 2004b) and sustain highly diverse communities of associated organisms (Jackson et al. 2003, Wilson et al. 2004). In a rhodolith bed, aggregations of live rhodoliths up to several cm deep can be found atop layers of dead rhodoliths and rhodolith fragments, descending into sediment (Adey 1970, Foster et al. 2013).

Many invertebrates live inside rhodoliths or burrow in surrounding sediments (Kamenos et al. 2004a,c, Hinojosa‐Arango et al. 2014). Similarly, intertidal and subtidal crustose coralline algae (CCA) can play host to a variety of grazing and burrowing infauna (Adey and Hayek 2011, Chenelot et al. 2011, Adey et al. 2013) Thicker crusts host a greater diversity of infauna (Steneck and Paine 1986), including both calcifying and noncalcifying animals. The presence of an infaunal community structurally weakens the algal thallus (Steneck and Paine 1986, Adey and Hayek 2011) and may thus exacerbate potential effects of OA on structural integrity of thick coralline algal crusts. For example, thicker coralline crusts may be more vulnerable to the effects of OA (McCoy 2013, McCoy and Ragazzola 2014), and there is evidence that structural properties including cell wall thickness (Kato et al. 2014, McCoy and Ragazzola 2014) and load‐bearing strength (Ragazzola et al. 2012) will be affected.

A high cover of nongeniculate coralline algae, typically found under intense grazing, such as in an urchin barren under the grazed kelp canopy (Adey 1970), may inhibit the recruitment of other organisms (Breitburg 1984). Like recruitment enhancement, recruitment deterrence is a species‐specific effect. Some coralline algae inhibit barnacles and filamentous diatoms from recruiting (Padilla 1981), while others inhibit different species of filamentous and fleshy algae (Masaki et al. 1981). A high‐density coralline algal carpet can inhibit the recruitment of sessile space occupiers, such as polychaetes, barnacles, amphipods, bryozoans, and algae, even when grazers are excluded (Breitburg 1984). Such recruitment inhibition is closely tied to competition for space; when nongeniculate coralline algae dominate the primary substrata, recruits of other sessile organisms must recruit onto the coralline algal thallus, where it is subsequently sloughed off along with the crustose coralline's uppermost layer of cells (Masaki et al. 1981).

Both rhodolith beds and coralline carpets can be important in the coastal carbon cycle in temperate areas. Carbonate accretion rates attributable to red coralline algae can vary between 79 and 1,432 g CaCO_3_ · m^−2^ · year^−1^ in North Atlantic rhodolith beds (Bosence 1980, Freiwald and Henrich 1994) and up to 1,350 g CaCO_3_ · m^−2^ · year^−1^ in geniculate coralline carpets the North Pacific (Fisher and Martone 2014). Because coralline algae are such important producers of CO_3_^2−^ (Adey 1965, Adey and Macintyre 1973, Basso 2011, Adey et al. 2013), the physical and chemical function of high‐latitude coralline algae in response to changes in the seawater environment will be an important area of study as changes to the marine environment continue. High‐latitude habitats have so far received little attention compared to warm‐water beds in this context.

## Photosynthesis and Calcification

### Growth

In coralline algae, growth characteristics depend on morphotype as well as the growth environment, specifically water motion, depth, and temperature. Among nongeniculate forms, Steneck (1985) found an inverse relationship between crust thickness and growth rate, which is hypothesized to be because thicker crusts maintain a greater quantity of living nonphotosynthetic tissue. An energetic trade‐off comes into play between lateral growth and maintenance of nonphotosynthetic tissue. Some species, therefore, form only thin crusts and have determinant vertical growth (Steneck and Paine 1986), whereas many others exhibit indeterminate vertical growth and form yearly or season growth bands (e.g., *Clathromorphum* spp., Adey et al. 2013). Nongeniculate coralline algae grow relatively slowly (vertically 0.3–10 mm · year^−1^, Setchell 1926, Adey and Vassar 1975, horizontally 0–10 mm · year^−1^, McCoy and Pfister 2014).

Geniculate and branched nongeniculate coralline algae do tend to grow faster (8–30 mm · year^−1^; Steneck and Adey 1976, Martone 2010) with no ontogenetic effect on growth rate (Fisher and Martone 2014). This is likely because geniculate and branched nongeniculate coralline algae have a greater photosynthetic capacity derived from the increased surface area of their branches. However, high latitude branched nongeniculate species can have growth rates as low as 200–300 μm · year^−1^ (Kamenos et al. 2008b), related to lower irradiance and colder water temperatures at high latitudes.

### Photosynthetic characteristics under natural conditions

Generally, temperate nongeniculate coralline algae are low‐light adapted (Burdett et al. 2012b), and exposure to higher light intensities causes a reduction in photosynthetic activity and bleaching of algal tissue, related to loss of photosynthetic pigments in surface cells (Irving et al. 2004, Martone et al. 2010a). This is not the case for tropical nongeniculate coralline algae that are found growing under high light levels on reef or algal ridge settings (Steneck and Adey 1976, Adey 1978, 1998), where they rely on dynamic photoinhibition to tolerate high photosynthetically active radiation (PAR; Burdett et al. 2014). In the temperate rhodolith *L. glaciale*, within‐thallus variability in light adapta‐tion has been documented, with branch bases less light‐acclimated than the tips (Burdett et al. 2012b). This may translate to differential light availability across the thallus in rhodolith beds. In addition, there is evidence for seasonal acclimation to differing light levels in summer and winter (Burdett et al. 2012b). Geniculate coralline algae show variation in light adaptation with zonation patterns across the intertidal and subtidal zones (Guenther and Martone 2014). Patterns in light tolerance and thereby coralline algal growth may be important to coastal carbon dynamics and thus important to document further across a range of species and environments.

### Calcification

Calcification rate in coralline algae is thought to be directly related to photosynthetic rate (Pentecost 1978), as well as to the ambient concentration of inorganic carbon when carbon availability is manipulated in a laboratory setting (Smith and Roth 1979, Gao et al. 1993). Evidence points to a “*trans* calcification” mechanism, as defined by McConnaughey and Whelan (1997) based primarily on the green freshwater alga *Chara corallina*, but documented in most biological calcification. In this mechanism, calcification is enzymatically driven; seawater HCO_3_^−^ is taken up and converted to carbon dioxide (CO_2_) for photosynthesis by an external carbonic anhydrase, which in turn produces the CO_3_^2−^ used in algal calcification (McConnaughey and Whelan 1997). Digby (1977) provided a more detailed mechanism developed for *Clathromorphum* and *Corallina* spp. based on pH drift and oxygen evolution measurements in the field and in the laboratory, suggesting that diffusion of hydrogen ions of the cell (most likely at the growing tips) promotes diffusion of seawater HCO_3_^−^ into the cell.

Although we understand some basic relationships between biological rates of growth (photosynthesis and calcification) and abiotic parameters such as temperature (e.g., Martin et al. 2007a,b, Burdett et al. 2012a,b), the ongoing foci on coralline algae under stress from global change and on integrating additional abiotic stressors will promote our increased understanding of these physiological processes as a function of environmental parameters. Geniculate coralline algae, for example in the genera *Amphiroa*,* Bossiella*,* Calliarthron*, and *Corallina*, are often used in growth experiments due to their higher growth rates compared to nongeniculate coralline algae, and thus much of our information about calcification in coralline algae comes from geniculate species. HCO_3_^−^ is the primary carbon species used in photosynthesis (Borowitzka 1981). In the geniculate alga *Corallina pilulifera*, calcification and photosynthesis increased in response to elevated dissolved inorganic carbon (DIC: CO_2(aq)_, HCO_3_^−^, and CO_3_^2−^), but not in response to addition of free CO_2_ (Gao et al. 1993). Inhibition of calcification at high seawater pH (>9) is most likely due to release of CO_2_ during respiration, which may cause localized acidification and reduced availability of DIC (Borowitzka and Larkum 1976). It is still unclear to what extent external carbonic anhydrase, an enzyme that enables algae to use HCO_3_^−^ for photosynthesis, is used throughout the coralline algae (Koch et al. 2013).

Evidence from the nongeniculate genus *Clathromorphum* suggests that at least some coralline algae can grow in extended periods of darkness (Adey 1998, Adey et al. 2013). These observations from field specimens contribute to our understanding of dark calcification, which otherwise comes from laboratory experiments conducted on 0–24 h timescales (Ikemori 1970, Pentecost 1978, Borowitzka 1979, Borowitzka and Larkum 1976, Smith and Roth 1979, Borowitzka 1981, El Haïkali et al. 2004). Dark calcification is likely sourced by an accumulation of energy during periods of light and photosynthesis, facilitated by the presence of secondary pit connections or cell fusions which are believed to allow for translocation of photosynthates within the coralline algal thallus (Pueschel and Cole 1982, Steneck 1983). This “accumulated energy” mechanism would also explain observed dissolution and restricted growth described under stressful conditions. Skeletal dissolution can occur in the dark even under ambient *p*CO_2_ concentration due to reduced pH in the diffusion boundary layer between the algal surface and surrounding seawater (Hurd et al. 2011). This can be tempered by an ability to compensate for *p*CO_2_‐induced nighttime dissolution by increasing their calcification rate during the day (Kamenos et al. 2013, Martin et al. 2013). Under elevated *p*CO_2_, however, increased photosynthesis is restricted during the day, and this reduces the capacity for enhanced daytime calcification (Kamenos et al. 2013). We point to a great need for (i) a better understanding of the function of secondary pit connections and cell fusions, including their role in calcification and growth, and (ii) molecular studies of up‐ or down‐regulation of enzymes used in both calcification and photosynthesis to establish a mechanistic molecular and biochemical understanding of calcification in corallines and the energetic requirements or trade‐offs associated with short‐ and long‐term calcification in the dark.

### Skeletal mineralogy and seawater conditions

All but three cell types are calcified in the coralline algae; (i) cells of reproductive structures, (ii) branch joints (genicula) of geniculate growth habits, and (iii) lesion sites of the thallus undergoing reparation (Borowitzka and Vesk 1978, Bilan and Usov 2001, Pueschel et al. 2005). CaCO_3_ composition of algal tissue thus varies by species, cell type, and the age of the alga (Borowitzka 1982). Coralline red algae (Corallinales and Sporolithales) are among only two known groups of marine algae, along with the family Coccolithaceae in the phylum Haptophyta (Guiry and Guiry 2014), that precipitate primarily the calcite rather than aragonite polymorph of CaCO_3_ (Bilan and Usov 2001). Coralline algae can precipitate aragonite as well as magnesium calcite, and this plasticity in skeletal composition is determined by the alga's local growth environment (Pueschel et al. 1992, Medakovic et al. 1995). Magnesium is part of the crystal lattice rather than being present in associated organics (Kamenos et al. 2009) and is thought to be incorporated secondarily into the crystal structure (Baas‐Becking and Galliher 1931). Calcite is deposited within the cell wall, and polysaccharide fibrils in the cell walls serve as a structural matrix for the formation of calcite tissues (Borowitzka 1982), with species that exhibit greater mineralization having lower polysaccharide content (Bilan and Usov 2001). The only differences between the ultrastructure and morphology of coralline red algae and other noncalcified red algae that may account for mineralization in this group are found in the polysaccharide composition of coralline algal cell walls (Bilan and Usov 2001).

Geniculate coralline algae thus present insight to the process of CaCO_3_ precipitation and nucleation in coralline algae. A case study of the geniculate *Calliarthron cheilosporioides* identified changes in the synthesized polysaccharides between genicula and intergenicula (Martone et al. 2010b). Specifically, xylogalactans, a type of agaran organic compound, form xylose side chains from the galactan backbone that act as nucleation points for CaCO_3_ precipitation. Within the genicula of *C. cheilosporioides*, these side chains are modified or absent, thereby controlling the location of calcification within the geniculate coralline algal thallus (Martone et al. 2010b). Calcification in geniculate coralline algae may be highly specified, as evidenced by cells at the geniculum–intergeniculum interface that are half‐calcified and half‐uncalcified (Johansen 1981, Martone et al. 2010b). Such interesting observations further illustrate the need for molecular mechanistic studies of calcification in the coralline algae.

Corallinales and Sporolithales exhibit a high degree of mineralogical variability, though most are calcitic (Smith et al. 2012). Mineralogy is generally related to growth habit, and geniculate coralline algae typically contain no aragonite (Smith et al. 2012). Mg^2+^ content is roughly phylogenetically variable (10.5%–16.4% by weight), with the Corallinaceae containing more Mg^2+^ than the Sporolithales and the Hapalidiaceae (Smith et al. 2012). Latitudinal trends can be used to explore effects of temperature gradients, though latitude is also coupled with irradiance (Halfar et al. 2000, 2011). Coralline algae are rare among mineralizing organisms in that they are able to respond to ambient seawater chemistry and change their skeletal mineralogy with seawater Mg^2+^ concentrations, although whether to reduce the energetic cost of mineralizing or as a response to chemical stress remains unknown (Stanley et al. 2002, Ries 2006a,b). Different coralline algae have been found to respond differently to nonpreferred seawater chemistries. *Neogoniolithon* and *Amphiroa* sp. were able to adjust to ambient seawater Mg concentrations in the laboratory, but with a loss of skeletal organization at low Mg^2+^/Ca^2+^ (Ries 2010). In other organisms (corals), undersaturation of a preferred skeletal mineral has induced thin or no skeletons (Fine and Tchernov 2007).

Mineralogy can show fine‐scale seasonal fluctuations in response to ambient water temperature (Darrenougue et al. 2013). More Mg^2+^ appears to be incorporated during faster growth (Moberly 1968, Kolesar 1978), which produces a relationship between Mg content and temperature (Chave and Wheeler 1965, Milliman et al. 1971). On the other hand, some recent studies indicate that replacement of Ca^2+^ by Mg^2+^ within the crystal lattice may be driven by temperature and not by growth rate (Kamenos et al. 2008b, 2009). Further work in this area is needed to separate the responses of temperature and growth rate and in particular to study species relationships between growth, temperature, and Mg content (sensu Adey and McKibbin 1970).

## Global Change Impacts on Physiology

### Elevated pCO_2_

Many previous studies on the effects of elevated *p*CO_2_ on coralline physiology and growth occurred prior to concerns over OA (e.g., Smith and Roth 1979, Borowitzka 1981, Gao et al. 1993). More recent work has extended physiological relationships with the higher *p*CO_2_ levels projected for future climate scenarios, and corroborates the previous foundational work that indicated a parabolic growth response to pH and *p*CO_2_ (Ries et al. 2009, Büdenbender et al. 2011). In the intertidal alga *Ellisolandia elongata*, for example, *p*CO_2_ was found to have no effect on respiration, gross primary production, and calcification rates in both light and dark (Egilsdottir et al. 2013, as *Corallina elongata*).

### Pollution

Effects of pollution from domestic sewage can cause increased turbidity and sedimentation of organic particles accompanied by eutrophication (Bell 1990). High levels of phosphate found in eutrophied areas have negative effects on growth and calcification in coralline algae (Björk et al. 1995). Phosphate inhibits calcite crystal growth by settling on the crystal surface, thereby preventing the formation of a crystal lattice that allows the crystals to grow (Simkiss 1964).

Herbicides have also been shown to have negative effects on photosynthesis in coralline algae. In particular, the marine herbicide diuron is used widely as an antifouling agent in marine environments and as an agricultural herbicide in the terrestrial environment (Hamilton and Haydon 1996, Martinez et al. 2001). The use of diuron in coastal tropical sugar plantations poses a real threat to coralline algae, which experience decreased photosynthetic activity in its presence (Harrington et al. 2005). This stress is exacerbated by sedimentation stress, which is also elevated in coastal agricultural areas (Harrington et al. 2005).

### Multiple stressors

Overall, in the face of multiple pressures from changes in climate and community reshuffling expected from range shifts of other algae, coralline algae are expected to become less widespread at high latitudes by the end of the current century, as illustrated by recent case studies in the North Atlantic (Brodie et al. 2014). Elevated temperature has been shown to act synergistically with elevated *p*CO_2_ to reduce tissue growth, though again much variation has been observed. In a study of the Mediterranean nongeniculate coralline *Lithophyllum cabiochae*, algal necroses were observed first in high temperature and the highest CO_2_ (700 ppm) treatment, followed by high temperature, 400 ppm CO_2_ treatments (Martin and Gattuso 2009). In *L. cabiochae*, dissolution rates exceed calcification only when both temperature and *p*CO_2_ were elevated, and dissolution rates were 2–4 times greater at elevated *p*CO_2_ (Martin and Gattuso 2009). In the rhodolith *Lithothamnion corallioides*, elevated temperatures reduced photosynthetic pigment content, whereas elevated *p*CO_2_ affected gross production and net calcification (Noisette et al. 2013a). These observations reveal important effects of both temperature and *p*CO_2_, though not directly acting together in all cases. Similar results have been found when elevated CO_2_ levels are combined with ultraviolet radiation (UVR), which can act synergistically with CO_2_ to affect photosynthesis, growth, and calcification (Gao and Zheng 2010).

Canopy cover in shallow coastal areas promotes the growth of coralline algal beds in the understory (Irving et al. 2004). Experimental reductions in canopy cover in both temperate and polar regions lead to crust bleaching as a result of increased PAR and UVR (Irving et al. 2004, 2005). In coralline algae, bleaching is defined as loss or degradation of photosynthetic pigments in surface tissue, such that the affected area appears white. In intertidal zones, however, coralline bleaching seems to be most strongly induced by desiccation stress, which can be tightly coupled to high temperature and light stress at low tide (Martone et al. 2010a), in addition to high irradiance and low canopy cover (Irving et al. 2004). However, because light and temperature alone had only mild effects on loss of pigmentation in the intertidal *Calliarthron tuberculosum*, it has been hypothesized that desiccation is responsible for coralline algae living above the low intertidal zone occurring primarily in tide pools (Martone et al. 2010a). Anecdotally, bleaching can be reversible in some situations, typically depending on the duration and severity of environmental stress (S.J. McCoy pers. obs.). In other cases, nongeniculate coralline individuals may overgrow their own bleached tissue. For example, *Pseudolithophyllum neofarlowii* lives in the upper intertidal zone on vertical surfaces in the Northeast Pacific. This species can be recognized by the texture of its thallus, which is comprised of many small protuberances (Steneck and Paine 1986) that serve the dual function of protecting live tissue beneath and flaking off easily to allow for new growth (S.J. McCoy pers. obs., R.T. Paine personal communication). Clearly, the long‐term effects of coralline algal bleaching and the different factors contributing to the reversible or irreversible nature of bleaching (e.g., pigment loss vs. pigment degradation) are areas where additional investigation is needed.

### Variable conditions

Recent coastal pH data sets reveal large diurnal fluctuations in photosynthesis‐dominated ecosystems that include tropical reefs as well as large expanses of temperate coastal areas where coralline algae are abundant (Wootton et al. 2008, Delille et al. 2009, Semesi et al. 2009, Anthony et al. 2011, Kleypas et al. 2011, Wootton and Pfister 2012, Cornwall et al. 2013a). *Porolithon onkodes* individuals sampled from a naturally variable environment calcified 42% more in variable *p*CO_2_ conditions than individuals from a uniform environment (Johnson et al. 2014). Interestingly, individual acclimation did not reduce the detrimental effects of exposure to a high *p*CO_2_ treatment (660 μatm), which decreased calcification by at least 70% in all individuals (Johnson et al. 2014). In contrast, pH manipulation to mimic diurnal fluctuations in kelp forest systems reduced growth rates of the geniculate coralline alga, *Arthrocardia corymbosa*, at lower pH. Growth was further reduced additively by pH fluctuation, though recruitment, and elemental composition of algal tissue did not change with pH (Cornwall et al. 2013b).

### Generalizations across morphologies and environments

Noisette et al. (2013b) showed that the metabolic rates of coralline algae across three growth forms, rhodolith (*Lithothamnion corallioides*), nongeniculate (*Lithophyllum incrustans*), and geniculate (*Corallina elongata*), vary in response to increasing seawater *p*CO_2_. However, only one species of each growth form was studied, making it difficult to determine whether these metabolic responses represent differential responses across species or across morphotypes. Mechanisms of skeletal response to pH vary by morphological and growth type of nongeniculate species. Comeau et al. (2014) found variable, location‐specific responses to elevated *p*CO_2_ in the nongeniculate coralline *Porolithon onkodes* across sites that differ in environmental conditions and carbon chemistry across the tropical Pacific, showing yet another degree of response variability. In another example, thick, slow‐growing species reduced their thallus thickness while keeping skeletal density and cell wall thicknesses constant (McCoy 2013, McCoy and Ragazzola 2014). In contrast, thin, fast‐growing species showed no change in thallus thickness, but instead reduced the thickness of interfilament cell walls (McCoy and Ragazzola 2014). This mechanism may reduce the amount of CaCO_3_ required for rapid lateral growth in species with this growth strategy.

Small physiological or morphological differences between species may therefore translate to changes in population and community ecology, as has already been shown in communities of nongeniculate coralline algae (McCoy and Pfister 2014, Ordoñez et al. 2014). Clearly, more physiological studies of responses to climate stressors are needed across growth forms, preferentially replicated across phylogenetic relationships. We note here that recent molecular advances have allowed a more precise study of coralline algal taxonomy and phylogenetics (Bailey and Chapman 1998, Le Gall and Saunders 2007, 2010, Broom et al. 2008, Bittner et al. 2011, Gabrielson et al. 2011, Kato et al. 2011, Martone et al. 2012, Hind and Saunders 2013, Hind et al. 2014), and we expect many more changes in coralline algal phylogeny as more groups are sequenced in the near future.

Long‐term studies can reveal markedly different results than shorter term studies. For example, laboratory cultures of *L. glaciale* maintained growth rates while decreasing skeletal quality (intra‐ and intercellular wall thicknesses) after exposure to acidification for 3 months (Ragazzola et al. 2012). After exposure to acidification for 10 months, the opposite result was observed; *L. glaciale* cultures preserved skeletal quality and reduced growth rates (Ragazzola et al. 2013). This is a classic example of energetic trade‐offs, where plants alter their resource allocation patterns differently to cope with short‐term compared to long‐term stressors (Grime 1979). More generally, documented effects of coralline algal bleaching, tissue necrosis, and reduced thallus thickness include weakened structural integrity (Ragazzola et al. 2012) that may lead to increased susceptibility of coralline algal beds to physical disturbances (Martone et al. 2010a, Egilsdottir et al. 2013, McCoy 2013).

The importance of conducting experiments in an ecological context is becoming increasingly apparent (Fisher and Martone 2014, McCoy and Pfister 2014). Recent measurements of net primary productivity of a pH gradient of 7.9–8.1 along the Oregon (USA) coast showed a reduction in coralline (*Corallina vancouveriensis*) productivity with lower pH, but a neutral effect of reduced pH on productivity of a coralline‐kelp assemblage (*C. vancouveriensis* and *Saccharina sessilis*; Tait 2014). Previous work with whole‐lake acidification experiments has taught us that interactions between an entire species assemblage, as well as between biological and geochemical processes, cannot be simulated in a laboratory study (Schindler 1990). Yet, they play crucial roles in ecological responses to perturbations. It is important that future ecological work exploring effects of OA, temperature, and other stressors on coralline algae take into account the natural context of those responses.

## Paleoenvironmental Recorders

### Paleoecological proxies

Paleoecological studies in shallow marine environments focus on the reconstruction of ecological communities or coastal environmental characteristics, typically by identifying species with known environmental tolerances or ecological functions in fossil assemblages or sediment cores (Adey and Steneck 2001, Perry and Hepburn 2008). In this context, coralline algae are typically used in the reconstruction of tropical CO_3_^2−^ environments. Combined with sediment analyses, the development of coastal reefs or shallow marine communities (e.g., Macintyre and Glynn 1976, Martindale 1992, Webster and Davies 2003, Payri and Cabioch 2004, Tierney and Johnson 2012) and community recovery from disturbance events (e.g., Perry 2001, Toth et al. 2012) can also be studied over time. From a more geological perspective, such reconstructions can also provide climatic context in which sediments were laid down (Braga and Aguirre 2001). Species' depth distributions, for example, can be used to reconstruct changes in sea level or reef accretion at a given locality (e.g., Cabioch et al. 1999, Yamano et al. 2001). Coralline algal ridges (also termed bioherms or mounds) provide a particularly accurate estimate of sea level, as they are restricted to the wave crest zone, and can track sea level within 10 cm (Adey 1986).

### Ultrastructure and growth banding

**Figure 3:**
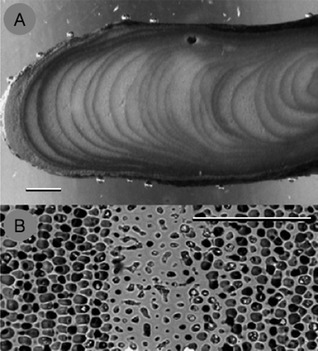
(A) Transverse section through *Lithothamnion glaciale* branch tip showing seasonal banding patterns (scale bar = 500 μm). (B) SEM micrograph showing cell structure of growth banding (scale bar = 100 μm). Cells with lower calcite density (%) deposited at warmer temperatures (left and right sides of B), created seasonal banding structure observed at lower magnification in A. Modified from Kamenos et al. 2008b.

The ultrastructural and mineralogical responses of coralline algae to ambient environmental conditions enable them to act as paleoenvironmental proxies, with the longest temperature reconstruction extending over 650 years (Kamenos 2010). While growing, rhodoliths and CCA of indeterminate thickness lay down annual and subannual growth bands composed of high‐Mg calcite (Fig. 3; Henrich et al. 1996, Kamenos et al. 2008b). In some species, growth bands are annual (*L. glaciale*, Kamenos et al. 2008b and *Clathromorphum compactum*, Halfar et al. 2008) while in other species, for example, *Phymatolithon calcareum*, subannual banding is present (Blake and Maggs 2003). Information on past climatic regimes can be locked within such growth bands either as structural or geochemical information.

Algochronology is the use of structural metrics obtained from the growth bands of coralline algae to determine past environmental conditions (Kamenos and Law 2010). Rhodoliths lay down regular annual, and or subannual, growth bands. The frequency of the bands has been reviewed by in depth by Foster (2001). Many encrusting species do not show growth banding, likely because of high grazing pressure and a set vertical thickness in those forms. Similarly, geniculate species do not form growth bands, because they grow primarily by forming new apical segments. In rhodoliths, bands can be formed due to reduced light availability and lower temperature during winter, reduced water movement, burial, monthly/lunar growth cycles driven by tidal patterns and possible large scale climate patterns, for example, El Niño (Foster 2001). The banding patterns are created by changes in the degree of cellular calcification (Fig. 3); in *L. glaciale* a negative correlation exists between calcite density of calcified cells and temperature (Kamenos 2010) as well as temperature and light availability (Burdett et al. 2011). Growth banding patterns can therefore be used to infer environmental processes in historical climate reconstructions.

While the width of the bands themselves do not appear to be well correlated to environmental conditions in *L. glaciale* (Kamenos and Law 2010, Burdett et al. 2011), by averaging the growth bands of multiple *Clathromorphum compactum* or *Clathromorphum nereostratum* thalli, this generated positive relationships with instrumental sea surface temperature (SST), enabling reconstruction of SST (Halfar et al. 2010, 2011). Significantly stronger relationships are present between calcite density and environmental conditions with negative relationships being present between temperature and calcite density in *L. glaciale* (Kamenos and Law 2010) as well as temperature and light (as PAR), enabling the reconstruction of both temperature and cloud cover (Burdett et al. 2011). Ultrastructural comparisons with a single environmental parameter can be characterized by noticeable variability (possibly caused by localized irradiance differences) but, by conducting calibrations using both temperature and light, that variability can be minimized (Burdett et al. 2011).

### Geochemistry

The CaCO_3_ skeleton of coralline algae contains multiple elements and their isotopes whose concentrations have been used in paleoenvironmental reconstruction. First insights into their geochemical elemental responses were made in the 1960s (Chave and Wheeler 1965, Moberly 1968). More recently, both elemental and isotopic deviations within their skeletons have been used as paleoclimate proxies.

### Diagenetic effects and proxy development

Coralline algae are protected by the presence of a living epithallium covering the CO_3_^2−^ skeleton often minimizing diagenetic effects (Alexandersson 1974). There is no evidence of unquantified vital effects, or altering of the chemical composition by the algae, in Mg/Ca temperature relationships (Kamenos et al. 2008b). Carbon and oxygen isotope ratios can be affected by the calcification process in many calcareous algae (Codiaceae, Daycladaceae, Corallinales) caused by kinetic fractionation associated with CO_2_ hydroxylation during calcification (Lee and Carpenter 2001). Some studies find an offset from isotopic equilibrium (~3.5‰; e.g., Halfar et al. 2000, Lee and Carpenter 2001, Williams et al. 2011) while in other in other studies, no evidence of isotope disequilibrium is observed (Rahimpour‐Bonab et al. 1997). The calcification and fractionation process may therefore be somewhat variable within the Corallinales or perhaps from one locality to another, and therefore calibration or validation is recommended.

Before using coralline algae as proxies for new variables, it is important that a three‐step process is followed to ensure the accuracy and precision of the reconstruction (Kamenos et al. 2009): (i) Calibration of a particular species to see if a relationship between the environmental variable and within‐algal proxy is present; (ii) validation of the relationship using biogeochemical analyses (e.g., molecular level characterization via synchrotron) to determine if the observed response meets the geochemical assumptions on which the proxy has been developed (e.g., Ca^2+^ substitution by Mg^2+^ ions in the calcite lattice at higher temperatures (Oomori et al. 1987, Kamenos et al. 2009); and (iii) application of that species as a calibrated and validated proxy.

### Magnesium (Mg)

**Figure 4:**
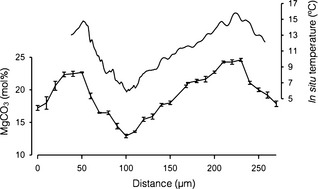
Mean MgCO_3_ ± SD in mol % shown by solid black line with error bars. Measurements made along transverse section (from apex to base of branch) of *Lithothamnion glaciale* by electron microprobe analysis. Solid black line with no error bars shows in situ temperature at time of skeletal deposition. Reproduced from Kamenos et al. 2008b.

Mg concentrations in biogenic CaCO_3_ have a positive relationship with temperature and are the most commonly used proxy. Mg concentrations, as Mg/Ca or MgCO_3_, have been calibrated and validated as in situ temperature proxies in nongeniculate coralline algae (Kamenos et al. 2008b, 2009) and have been used to reconstruct marine temperature from fortnightly to decadal resolution (Fig. 4). In the northern hemisphere, these species are *L. glaciale* (Halfar et al. 2000, Kamenos et al. 2008b, Kamenos 2010, Kamenos et al. 2012), *Lithophyllum kotschyanum* (Caragnano et al. 2014), *Lithothamnion crassiusculum* (Halfar et al. 2000), *Phymatolithon calcareum* (Kamenos et al. 2008b), *Clathromorphum nereostratum* (Hetzinger et al. 2009, 2012, Williams et al. 2011), and *Clathromorphum compactum* (Gamboa et al. 2010, Hetzinger et al. 2012); in the Southern Hemisphere *Sporolithon durum* (Darrenougue et al. 2013).

### Trace elements: Barium (Ba), Lithium (Li), Strontium (Sr), Uranium (U)

Ba/Ca in nongeniculate coralline algae have been used to reconstruct seas surface salinity in Atlantic Canadian *Clathromorphum compactum* (Hetzinger et al. 2013), in Alaskan *Clathromorphum nereostratum* (Chan et al. 2011) and in Yemeni *Lithophyllum kotschyanum* from nutrient rich upwellings (Caragnano et al. 2014) at subannual resolutions, but all studies found no relationship with temperature. Li/Ca in Yemeni *Lithophyllum kotschyanum* at subannual resolution has been used to reconstruct temperature (Caragnano et al. 2013).

While attempts have been made to use Sr concentrations for temperature reconstruction, they appear to be strongly influenced by vital effects or kinetic incorporation of Sr ions into the calcite lattice of *Sporolithon durum* (Darrenougue et al. 2013), *L. glaciale*,* Phymatolithon calcareum* (Kamenos et al. 2008b), and also *Clathromorphum compactum* (Hetzinger et al. 2011). Incorporation of U in *Clathromorphum compactum* was not found to be influenced by temperature (Hetzinger et al. 2011).

### Isotopes

In nongeniculate coralline algae, the stable isotopic ratio of oxygen (reflecting incorporation of ^16^O vs. ^18^O), δ^18^O, records both temperature and salinity (Halfar et al. 2000, Kamenos et al. 2012). δ^18^O has been used to reconstruct temperature using *L. glaciale* (Halfar et al. 2000, 2007), *Lithothamnion crassiusculum* (Halfar et al. 2000), and by subtraction of the temperature component of the signal, salinity in *L. glaciale* (Kamenos et al. 2012).

Changes in the stable isotopic ratio of carbon (incorporation of ^12^C vs. ^13^C), δ^13^C, have been used to reconstruct DIC concentrations in *Clathromorphum compactum* (Williams et al. 2011), and ^14^C concentrations have been used to determine long‐term growth rates of *Clathromorphum nereostratum* (Frantz et al. 2005) and to date the exact timing of climatic events recorded by *Lithothamnion crassiusculum* (Frantz et al. 2000) and *L. glaciale* (Kamenos 2010).

### Associated variables, patterns, and ecosystem changes

Reconstruction of individual climatic parameters has also been used to understand changes in larger climatic phenomena. These include cloud cover via changes in cell size (Burdett et al. 2011), runoff from the Greenland Ice Sheet via changes in Mg/Ca and δ^18^O (Kamenos et al. 2012), Aleutian Low Pressure index via changes in band width (Halfar et al. 2011), North Atlantic Oscillation index via changes in Mg/Ca (Hetzinger et al. 2012), Atlantic Multidecadal Oscillation index via changes in Mg/Ca (Kamenos 2010), and Decadal Sea Level pressure via changes in Mg/Ca (Hetzinger et al. 2012).

Environmental reconstructions from nongeniculate coralline algae have proved useful in understanding how past changes in marine productivity relate to historic environmental change. In the north Atlantic, Mg/Ca temperature reconstructions from *L. glaciale* were negatively related to abundances of the copepod *Calanus finmarchicus* allowing a projection of copepod abundance to 2040 (Kamenos 2010). In the Bering Sea, growth increments in *Clathromorphum compactum* were used to understand landings in Sockeye Salmon via reconstruction on the Aleutian Low climate pattern (Halfar et al. 2011). The novel use of coralline algal proxies combined with ecological metrics in these studies indicates the potential of coralline algae for understanding the past and future drivers of marine productivity in addition to environmental change.

### Looking ahead

Research on coralline algae, particularly in the context of global climate change, has recently expanded among physiologists, ecologists, and geologists. In this review, we have summarized what is known in these areas in an effort to increase the accessibility of previous work on coralline algae for interdisciplinary researchers. In doing so, we have identified the following areas of need:

1. Molecular studies of algal calcification;
2. Resolution of the monophyly of genera and their phylogenetic relationships;
3. Potential generalization of physiological parameters to morphological or phylogenetic groups;
4. The role of multiple stressors on physiology, with an emphasis on integrating studies of pollutants;
5. Increased long‐term studies focusing on acclimatization potential to OA, temperature, and UV;
6. The mechanics and long‐term repercussions of coralline algal bleaching;
7. Effects of species‐level stress responses on local communities;
8. Community‐scale responses and field experiments;
9. Impacts of climate change on chemical cues;
10. Importance of coralline algae to reef stabilization under “future” scenarios; and
11. Refinement of coralline algae as paleorecorders with focus on the development of new proxies.

Coralline algae are a unique group of organisms in the context of global climate change. As photosynthesizers, calcifiers, ecologically important species, and paleoclimate archives, they enable us to ask diverse questions across the fields of phycology, physiology, ecology, geology, and conservation that will promote and require interdisciplinary cooperation.

We would like to thank CA Pfister for organizational comments on this manuscript and CC Stepien and PW Tierney for thoughtful discussions on this topic. The comments of our editor, PW Gabrielson, and several reviewers contributed greatly to this review SJM received fellowship support from a US NSF Graduate Research Fellowship, a DoD Air Force Office of Scientific Research National Defense Science and Engineering Graduate Fellowship, and the ARCS Foundation. NAK was funded by Natural Environmental Research Council UK grant NE/H010025.
